# Trends of Fatal Road Traffic Injuries in Iran (2004–2011)

**DOI:** 10.1371/journal.pone.0065198

**Published:** 2013-05-28

**Authors:** Ayad Bahadorimonfared, Hamid Soori, Yadollah Mehrabi, Ali Delpisheh, Alireza Esmaili, Masoud Salehi, Mahmood Bakhtiyari

**Affiliations:** 1 Department of Epidemiology, School of Health, Shahid Beheshti University of Medical Sciences, Tehran, Iran; 2 Safety Promotion and Injury Prevention Research Centre, Shahid Beheshti University of Medical Sciences, Tehran, Iran; 3 Department of Clinical Epidemiology and Prevention of Psychosocial Injuries Research Centre, Ilam University of Medical Sciences, Ilam, Iran; 4 Road Traffic Departments, Police Sciences University, Tehran, Iran; 5 Department of Statistics and Mathematics, School of Management and Medical Information, Tehran University of Medical Sciences, Tehran, Iran; 6 Department of Epidemiology and Biostatistics, School of Health and Health Research Institute, Tehran University of Medical Sciences, Tehran, Iran; The Australian National University, Australia

## Abstract

Road traffic injuries (RTIs) leading to death need the most essential concern for low, middle and high income societies. Mortality rate due to traffic injuries is considerable in Iran particularly during the last decade along with the industrialization process. The present study considered the trend of traffic injuries leading to death in Iran for a period of seven-years which started from March 2004 to March 2011. The formal merged Iranian database provided by the Ministry of Roads, the Legal Medicine Organization, the Traffic Police (NAJA), and the Ministry of Health covering 146, 269 deaths due to traffic injuries between 2004 and 2011 was analyzed. The time series method was carried out to determine the death trends of RTIs in the whole country. The Poisson regression model was used to estimate the changes in the frequency of events over time adjusting for associated known risk factors. The SARIMA (0, 1, 1)×(0, 1, 1)_12_ model was used for fitting to the time series of death rate. The death rate due to RTIs in Iran has statistically declined from 38 in 2004 to 31 per 100,000 populations in 2011. Based on the number of vehicles, the mortality rate has also declined from 38 to 12 cases per 10,000 vehicles from 2004 to 2011 respectively. However, the mortality rate was increased from 51 to 65 cases per 1000 accidents from 2004 to March 2011 respectively. Despite minor variations in mortality trends of RTIs in Iran according to different criteria, an annual average of 21,000 deaths is considerable and needs serious attentions. Modification of traffic laws, enhancement of police controls, improving transport infrastructure, holding education courses for drivers and providing optimal healthcare services are recommended.

## Introduction

Road traffic injuries (RTIs) refer to unexpected and unforeseen events involving at least one motor vehicle [1]. Fatal RTIs refer to the process whereby life is lost during accidental time or a maximum of 30 days after crash [2]. Deaths, injuries and disabilities resulting from RTIs are considered as the major concerns in public health [3], [4], [5]. RTIs are the main causes of death, disability and hospitalization and it also caused enormous socioeconomic burden [6].

It is anticipated that by 2020, deaths resulting from RTIs will be the third and second leading cause of death in high income and middle/low income countries respectively [7]. According to the World Health Organization, if appropriate action is not taken to reduce traffic accident by the end of 2020, deaths resulting from these accidents will increase by 67% [3]. However, the pattern of road traffic accidents and deaths are different in developed and developing countries which require specific approaches and strategies [8], [9], [10].

Deaths caused by RTIs should be of a particular concern in Iran [11], where only in 2007, 27567 people mostly the young and children lost their lives prematurely and 276762 were injured [12]. Therefore, reducing mortalities due to road traffic accidents should be the most important priorities for the government and health care system in particular [13]. The present study investigated the trends of RTIs leading to death in Iran for a period of seven-years which started from March 2004 to March 2011.

## Methods

Through a cross-sectional study, all police records of RTIs leading to death in Iran from March 2004 to March 2011 which were registered at the Information and Communication Technology (ICT) database (equivalent to the Persian abbreviation of FAWA) were investigated. This database was also approved by the Ministry of Road, the Legal Medical Organization, the Traffic Police and the Ministry of Health using the SQL software to be assured of the quality of data in order to refine and eliminate duplicate cases. Duplicates were removed in terms of three components of series of code names, serial number and date of accident.

According to the International Classification of Diseases and Causes of Death (ICD 10), traffic accidents are classified under the V01–V99 codes. In the present study, land road traffic accidents involving at least a motor vehicle with two wheels were included. Cases or events that were lacking these conditions were excluded from the study.

All analysis was done by SAS (9.2) and RGUI software. The time series analysis including Box and Jenkins was used to determine death trends in the whole country. The Poisson regression model was also adopted to estimate the frequency of events over time and to determine associated risk factors.

The study proposal was approved by the Shahid Beheshti University of Medical Sciences Ethical Committee and the Safety Promotion & Injury Prevention Research Centre, Tehran-Iran.

## Results

During the study period from March 2004 to March 2011, the Iranian population and the number of new vehicles have increased by 6,388,500 people and 11,961,947 vehicles respectively. However, the number of road traffic accidents and deaths were not uniform over the 7-year period (Table 1). The number of vehicles in Iran has been on the increase from 100 in 2004 to 251 in 2011 per 1000 populations. Overall, the number of deaths that occurred from traffic accidents in that period of time was up to 173, 834 cases. The frequency of RTIs increased from 510,000 in 2004 to 722,000 in 2006. However, this increasing figure decreased to 673,000 in 2007 and to 664,000 cases in 2008. Interestingly, death rate from road traffic accidents has been on the decline from 38 per 100,000 populations in 2004 to 31 per 100,000 in 2009 affecting the annual trends (Table 2).

**Table 1:** Distribution of RTIs leading to death in Iran.

Year	Population(n)	Vehicles(n)	RTIs (n)	RTIs/100,000 population (n)	RTIs/10,000 vehicles (n)	Deaths (n)
2004	68,344,730	6,797,623	510340	747	751	26089
2005	69,390,405	8,606,582	574511	828	668	27677
2006	70,495,782	10,476,807	721926	1024	689	27565
2007	71,532,063	12,511,460	672515	940	538	22918
2008	72,583,587	14,370,511	663673	914	462	23362
2009	73,650,566	16,389,240	511564	695	312	22974
2010*	74,733,230	18,759,570	414161	554	221	23249

RTIs: Road Traffic Injuries.

* Including data until March 2011.

**Table 2:** Mortality rate due to RTIs in Iran.

MR/1000 accidents	MR/10,000 vehicles	MR/100,000 populations	Year
51.1	38.4	38.2	2004
48.2	32.2	39.9	2005
38.2	26.3	39.1	2006
34.1	18.3	32.0	2007
35.2	16.3	32.2	2008
44.9	14.0	31.2	2009
56.1	12.4	31.1	2010*

MR: Mortality rate.

* Including data until March 2011.

**Figure 1:**
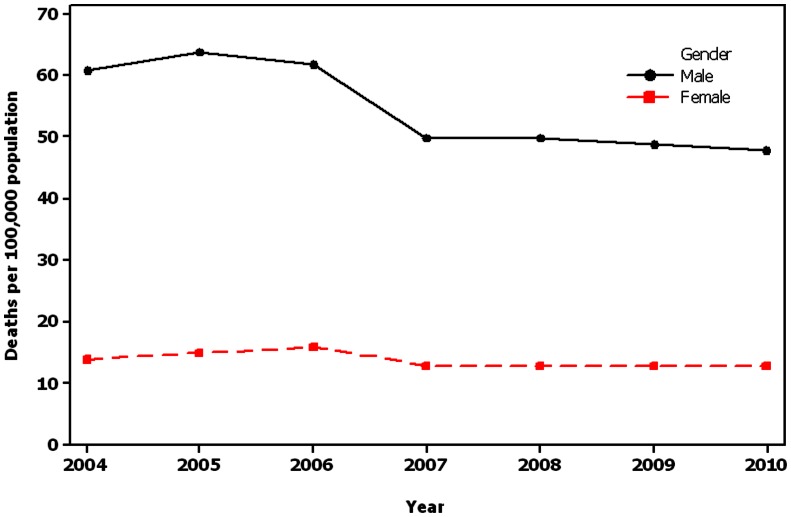
Trends of death rate per 100,000 populations in Iran by gender.

Male/female sex ratio for death rate due to RTIs in the present study was almost 4 to 1. Even though, the death rate has decreased in men but that is not the case in women, which remained constant after 2007 after severe increase between 2004 and 2006 (Figure 1).

**Figure 2:**
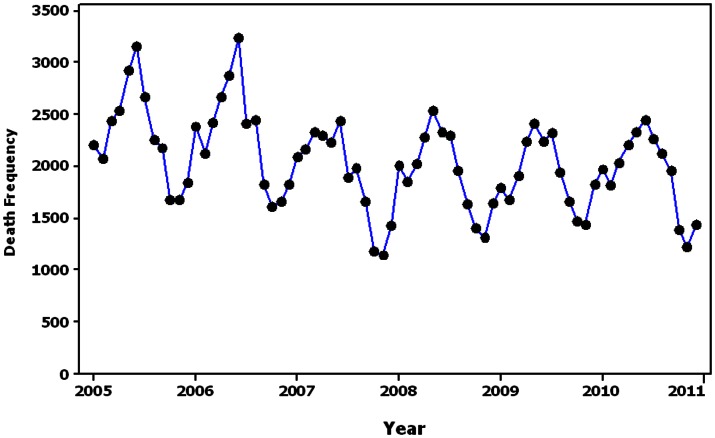
Time trends of the deaths from RTIs in Iran.

The time series analysis showed a seasonal pattern for RTIs leading to death in Iran in which the frequency of such events was significantly increased during the summer holidays. The value of the series was also decreased by increasing time on average (Figure 2).

The index values of AKAIKE Information Criterion (AIC) and SCHWARTZ Bayesian Criterion (SBC) for the estimation of Auto-Regressive Integrated Moving Average (ARIMA) models are shown in Table 3. The lowest AIC (790) for the model was the Seasonal Auto-Regressive Integrated Moving Average (SARIMA, (0,1,1)×(0,1,1)12, which is suitable for fitting into the series. Investigating auto-correlated residual after fitting model showed a tendency to zero on correlation between residuals and diagrams.

**Table 3:** Summary of time series analysis models.

Characteristics	Differencing	
	Seasonal	Seasonal & non-seasonal
Fixed estimate	−3.29	−0.76
Variance estimation	43253.70	33191.19
SE of estimate	207.97	182.18
AIC*	803.47	790.28
SBC**	807.62	796.52
Residuals (n)	59	59

* AKAIKE Information Criterion.

** SCHWARTZ Bayesian Criterion.

SE: Standard Error.

## Discussion

The present study is unique in terms of quality and quantity of data as it was prepared and confirmed by main relevant governmental organizations after merging data and deleting the repetitions. Therefore, the validity, reliability and representativeness of the analyzed data are assured. The total population of Iran has increased by 9% during the study period and the number of vehicles has almost been tripled. Increasing number of vehicles along with industrialization process in developing countries is expected. The number of vehicles in China between 2003 and 2005 had an average annual growth of 17.9% and from 62 to 100 vehicles per 1,000 populations which is almost equal to the statistics provided in the present study for the same number of years [14].

Comparison of RTIs per 100,000 populations in Iran (747), Azerbaijan Republic (28), Kazakhstan (94), Turkmenistan (28), Turkey (94), Australia (534), United States (675), and UK (370) implies a need for huge attention and consideration in Iran [15]. Reforming traffic regulations, increasing fines, adopting police and policy makers in order to comply with regulations and improving public health education could be considered as effective ways to reduce road traffic accidents in Iran. However, the roles of socio-cultural aspects should not be ignored. Although, the Iranian culture may be somewhat different from that of the European and other industrialized societies, even though the incentives to behaving well are often better than punishment of bad behavior in general. Laws should be followed but that is far from the only way to achieve safety. Nevertheless, better safety is primarily a result of building an environment where people behave responsibly without enforcement.

The RTIs related death in Iran has increased during 2004 to 2006, but declined in 2007. An evaluation of the trends of the death rate per population also confirmed this finding; Due to a significant reduction in 2007 compared to previous years could be partly due to changes in traffic regulations and driving policies starting from 2005.

Similar Iranian studies have shown that the death rate was 22 cases in 1997 and it has annually increased to 39 cases per 100,000 populations until 2010 [11], [16]. It has also been revealed that low and middle income countries had the highest mortality rate due to traffic accidents compared to developed countries (21.5 vs. 19.5 per 100,000 population respectively), [2], [17], [18]. In a Chinese study, a significant decrease was reported for death rate due to road traffic accidents from 8 in 1994 to 1 in 2005 [14], but not in Shanghai where the rate was up to 14 cases [19].

In the present study, males were mostly killed through road traffic accidents than females as similar to other studies [18]–[21]. This could be partly due to greater population at risk and the fact that males have more cars when compared to females. Comparison the death rate per 100,000 populations between the two genders showed that the rate was almost identical for men but not for women in the first 3 years of study. However, it was faced with a discernible decline of 18% in 2007. This finding could be due to less cautious and paying attention to driving symbols and regulations by men than women. Even though, in the suburban routes (roads) because of men driving skills and having greater ability to react in dangerous situation, men were less likely to be killed than women. In a recent study, the RTIs mortality rates were 39 and 11 deaths per 100,000 populations for men and women respectively [19]. A similar male/female discrepancy ratio has been reported in Europe, USA and Japan [20].

In the current study, the mortality trend from RTIs was declining per 10,000 vehicles from 2004 to March 2011. The highest decrease occurred in 2007 which was significantly lower than the previous years. Another Iranian study [16] has confirmed increasing the trends of death from RTIs per 10,000 vehicles between 1997 (21.1) and 2002 (25.8). However, that figure was declined by 17 cases in 2006. Similarly, a downward trend for these variables was observed between 2004 to 2007 from 24.2 to 13.4 that already reported which is less than the values obtained in the present study (38 in 2004, and 18 in 2007) [8].

The death trend from RTIs has been downward from the year 2004 to 2007, but has been upward with a great reduction of 25.7% in 2006. The highest deaths rate was observed between 2009 and March 2011. The reason behind this reduction could be partly due to the implementation of new regulations for financial compensations paid by insurance companies. Another study in Iran [16] has demonstrated that the figure of 85 deaths per 1000 RTIs in 1997 dropped to 42 cases in 2004 and between 2005 to 2006, and has remained relatively constant which is similar to the results of the present study. However, non-consistence results are reported [22]. There are several causes and mechanisms that lead to crashes. Alcohol consumption may not be a wide problem in Iran due to religious beliefs and governmental norms, but falling asleep when driving may also be one of the causes, other causes can also be considered such as over speeding, distraction by using mobile phones and talking to or trying to impress passengers.

In conclusion, assessment of death trends caused by RTIs in Iran from March 2004 to March 2011 showed a reduction in death rate per number of vehicles and mortality per 100,000 populations since 2007. This finding depends on human, environmental and road related factors. Police enforcements in this regard include the provisions to deal seriously with traffic offenders, the mandatory usage of safety belts for car passengers and a motorcycle helmet, using of fixed and mobile speed cameras, creation or expansion of fixed and mobile police stations in the highways and country roads are highlighted. Safety training programs for drivers and implementation of social programs such as police co-workers from primary and secondary students should be performed. Identify accidental prone areas and efforts to rebuild roads and optimization are helpful. Vehicle safety standards, optimization of the pre-hospital medical services, increasing road ambulances and emergency stations on the road, promotion of emergency services and employing trained medical personnel are recommended.
